# 4-Nitro-2-{[(tricyclo­[3.3.1.1^3,7^]decan-1-yl)iminium­yl]meth­yl}phenolate

**DOI:** 10.1107/S1600536812012597

**Published:** 2012-03-28

**Authors:** Kwang Ha

**Affiliations:** a School of Applied Chemical Engineering, The Research Institute of Catalysis, Chonnam National University, Gwangju 500-757, Republic of Korea

## Abstract

The title compound, C_17_H_20_N_2_O_3_, is a Schiff base, which is found as a zwitterion in the solid state. The geometry around the iminium N atom indicates *sp* ^2^-hybridization. The zwitterion shows a strong intra­molecular N—H⋯O hydrogen-bond inter­action between the iminium N atom and the phenolate O atom.

## Related literature

For the crystal structure of 2-[(tricyclo­[3.3.1.1^3,7^]decan-1-yl­imino)­meth­yl]phenol, see: Fernández-G *et al.* (2001 ▶).
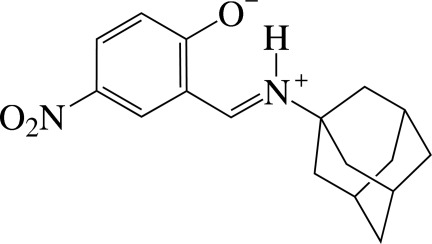


## Experimental

### 

#### Crystal data

C_17_H_20_N_2_O_3_
*M* *_r_* = 300.35
Triclinic, 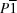

*a* = 6.3531 (5) Å
*b* = 11.0617 (10) Å
*c* = 12.1576 (11) Å
α = 62.995 (2)°
β = 76.446 (2)°
γ = 75.487 (2)°
*V* = 729.71 (11) Å^3^
*Z* = 2
Mo *K*α radiation
μ = 0.09 mm^−1^
*T* = 200 K
0.26 × 0.25 × 0.15 mm

#### Data collection

Bruker SMART 1000 CCD diffractometer
Absorption correction: multi-scan (*SADABS*; Bruker, 2000 ▶) *T*_min_ = 0.897, *T*_max_ = 1.000
4583 measured reflections
2822 independent reflections
1457 reflections with *I* > 2σ(*I*)
*R*_int_ = 0.029

#### Refinement

*R*[*F* ^2^ > 2σ(*F* ^2^)] = 0.053
*wR*(*F* ^2^) = 0.146
*S* = 0.95
2822 reflections
202 parameters
H atoms treated by a mixture of independent and constrained refinement
Δρ_max_ = 0.19 e Å^−3^
Δρ_min_ = −0.24 e Å^−3^

### 

Data collection: *SMART* (Bruker, 2000 ▶); cell refinement: *SAINT* (Bruker, 2000 ▶); data reduction: *SAINT*; program(s) used to solve structure: *SHELXS97* (Sheldrick, 2008 ▶); program(s) used to refine structure: *SHELXL97* (Sheldrick, 2008 ▶); molecular graphics: *ORTEP-3* (Farrugia, 1997 ▶); software used to prepare material for publication: *SHELXL97*.

## Supplementary Material

Crystal structure: contains datablock(s) global, I. DOI: 10.1107/S1600536812012597/fk2057sup1.cif

Structure factors: contains datablock(s) I. DOI: 10.1107/S1600536812012597/fk2057Isup2.hkl

Additional supplementary materials:  crystallographic information; 3D view; checkCIF report

## Figures and Tables

**Table 1:** Hydrogen-bond geometry (Å, °)

*D*—H⋯*A*	*D*—H	H⋯*A*	*D*⋯*A*	*D*—H⋯*A*
N1—H1*N*⋯O1	0.95 (3)	1.79 (3)	2.597 (3)	140 (2)

**Fig. 1.**
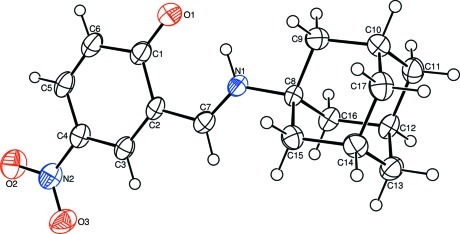
Molecular structure of the title compound, with atom numbering. Displacement ellipsoids are drawn at the 40% probability level for non-H atoms.

## supplementary crystallographic information

### Comment

The title compound, C_17_H_20_N_2_O_3_, is a Schiff base, which can act as a monobasic bidentate ligand, that is, the N,O donor atoms can coordinate to a metal ion. In the crystal structure, the Schiff base is found as a zwitterion (Fig. 1), whereas the closely related compound IDOHIS 2-[(tricyclo[3.3.1.1^3,7^]decan-1-ylimino)methyl]phenol with similar molecular geometry is in the phenol-imine form (Fernández-G *et al.*, 2001).

### Experimental

1-Adamantylamine (0.4556 g, 3.012 mmol) and 5-nitrosalicylaldehyde (0.5040 g, 3.016 mmol) in acetone (20 ml) were stirred for 3 h at room temparature. After addition of pentane (50 ml) to the reaction mixture, the formed precipitate was separated by filtration, washed with pentane, and dried at 50 °C, to give a yellow powder (0.8312 g). Crystals suitable for X-ray analysis were obtained by slow evaporation from a CH_3_CN solution at room temparature.

### Refinement

Carbon-bound H atoms were positioned geometrically and allowed to ride on their respective parent atoms: C—H = 0.95 — 1.00 Å with *U*_iso_(H) = 1.2*U*_eq_(C). Nitrogen-H atom was located from the difference Fourier map and allowed to refine with *U*_iso_(H) = 1.5 *U*_eq_(N).

### Crystal data

**Table:**

C_17_H_20_N_2_O_3_	*Z* = 2
*M**_r_* = 300.35	*F*(000) = 320
Triclinic, *P*1	*D*_x_ = 1.367 Mg m^−^^3^
Hall symbol: -P 1	Mo *K*α radiation, λ = 0.71073 Å
*a* = 6.3531 (5) Å	Cell parameters from 1063 reflections
*b* = 11.0617 (10) Å	θ = 3.4–24.9°
*c* = 12.1576 (11) Å	µ = 0.09 mm^−^^1^
α = 62.995 (2)°	*T* = 200 K
β = 76.446 (2)°	Block, yellow
γ = 75.487 (2)°	0.26 × 0.25 × 0.15 mm
*V* = 729.71 (11) Å^3^	

### Data collection

**Table:**

Bruker SMART 1000 CCD diffractometer	2822 independent reflections
Radiation source: fine-focus sealed tube	1457 reflections with *I* > 2σ(*I*)
Graphite monochromator	*R*_int_ = 0.029
φ and ω scans	θ_max_ = 26.0°, θ_min_ = 1.9°
Absorption correction: multi-scan (*SADABS*; Bruker, 2000)	*h* = −7→6
*T*_min_ = 0.897, *T*_max_ = 1.000	*k* = −13→13
4583 measured reflections	*l* = −13→14

### Refinement

**Table:**

Refinement on *F*^2^	Primary atom site location: structure-invariant direct methods
Least-squares matrix: full	Secondary atom site location: difference Fourier map
*R*[*F*^2^ > 2σ(*F*^2^)] = 0.053	Hydrogen site location: inferred from neighbouring sites
*wR*(*F*^2^) = 0.146	H atoms treated by a mixture of independent and constrained refinement
*S* = 0.95	*w* = 1/[σ^2^(*F*_o_^2^) + (0.0616*P*)^2^] where *P* = (*F*_o_^2^ + 2*F*_c_^2^)/3
2822 reflections	(Δ/σ)_max_ < 0.001
202 parameters	Δρ_max_ = 0.19 e Å^−^^3^
0 restraints	Δρ_min_ = −0.24 e Å^−^^3^

### Special details

**Table:**

Geometry. All e.s.d.'s (except the e.s.d. in the dihedral angle between two l.s. planes) are estimated using the full covariance matrix. The cell e.s.d.'s are taken into account individually in the estimation of e.s.d.'s in distances, angles and torsion angles; correlations between e.s.d.'s in cell parameters are only used when they are defined by crystal symmetry. An approximate (isotropic) treatment of cell e.s.d.'s is used for estimating e.s.d.'s involving l.s. planes.
Refinement. Refinement of *F*^2^ against ALL reflections. The weighted *R*-factor *wR* and goodness of fit *S* are based on *F*^2^, conventional *R*-factors *R* are based on *F*, with *F* set to zero for negative *F*^2^. The threshold expression of *F*^2^ > σ(*F*^2^) is used only for calculating *R*-factors(gt) *etc*. and is not relevant to the choice of reflections for refinement. *R*-factors based on *F*^2^ are statistically about twice as large as those based on *F*, and *R*- factors based on ALL data will be even larger.

### Fractional atomic coordinates and isotropic or equivalent isotropic displacement parameters (Å^2^)

**Table:**

	*x*	*y*	*z*	*U*_iso_*/*U*_eq_	
O1	1.0883 (3)	0.7989 (2)	−0.01074 (18)	0.0579 (6)	
O2	0.9391 (3)	0.5043 (2)	−0.3099 (2)	0.0681 (6)	
O3	0.6210 (3)	0.5069 (2)	−0.19649 (19)	0.0616 (6)	
N1	0.7093 (3)	0.7907 (2)	0.12908 (19)	0.0378 (6)	
H1N	0.856 (5)	0.809 (3)	0.103 (2)	0.057*	
N2	0.8088 (4)	0.5349 (2)	−0.2295 (2)	0.0487 (6)	
C1	1.0215 (4)	0.7417 (3)	−0.0628 (2)	0.0423 (7)	
C2	0.8046 (4)	0.7034 (2)	−0.0257 (2)	0.0357 (6)	
C3	0.7394 (4)	0.6358 (2)	−0.0807 (2)	0.0374 (6)	
H3	0.5980	0.6100	−0.0542	0.045*	
C4	0.8786 (4)	0.6058 (2)	−0.1734 (2)	0.0382 (6)	
C5	1.0878 (4)	0.6442 (3)	−0.2144 (2)	0.0438 (7)	
H5	1.1823	0.6230	−0.2787	0.053*	
C6	1.1554 (4)	0.7108 (3)	−0.1634 (2)	0.0463 (7)	
H6	1.2956	0.7382	−0.1946	0.056*	
C7	0.6570 (4)	0.7319 (2)	0.0713 (2)	0.0382 (6)	
H7	0.5154	0.7067	0.0939	0.046*	
C8	0.5734 (4)	0.8178 (2)	0.2350 (2)	0.0331 (6)	
C9	0.6942 (4)	0.9009 (3)	0.2633 (2)	0.0422 (7)	
H9A	0.8428	0.8495	0.2819	0.051*	
H9B	0.7103	0.9894	0.1898	0.051*	
C10	0.5661 (4)	0.9281 (3)	0.3747 (2)	0.0418 (7)	
H10	0.6461	0.9825	0.3931	0.050*	
C11	0.5428 (4)	0.7919 (3)	0.4882 (2)	0.0433 (7)	
H11A	0.6899	0.7389	0.5086	0.052*	
H11B	0.4611	0.8094	0.5610	0.052*	
C12	0.4202 (4)	0.7097 (2)	0.4600 (2)	0.0378 (6)	
H12	0.4043	0.6205	0.5345	0.045*	
C13	0.1928 (4)	0.7910 (3)	0.4292 (2)	0.0424 (7)	
H13A	0.1124	0.7370	0.4119	0.051*	
H13B	0.1078	0.8091	0.5011	0.051*	
C14	0.2172 (4)	0.9268 (2)	0.3153 (2)	0.0389 (7)	
H14	0.0683	0.9802	0.2954	0.047*	
C15	0.3463 (4)	0.8987 (3)	0.2030 (2)	0.0388 (7)	
H15A	0.2676	0.8451	0.1841	0.047*	
H15B	0.3608	0.9867	0.1287	0.047*	
C16	0.5489 (4)	0.6809 (2)	0.3483 (2)	0.0386 (6)	
H16A	0.6957	0.6266	0.3679	0.046*	
H16B	0.4699	0.6270	0.3300	0.046*	
C17	0.3388 (4)	1.0100 (3)	0.3438 (3)	0.0437 (7)	
H17A	0.2547	1.0292	0.4153	0.052*	
H17B	0.3536	1.0989	0.2707	0.052*	

### Atomic displacement parameters (Å^2^)

**Table:**

	*U*^11^	*U*^22^	*U*^33^	*U*^12^	*U*^13^	*U*^23^
O1	0.0459 (12)	0.0837 (15)	0.0525 (13)	−0.0252 (10)	0.0053 (9)	−0.0343 (12)
O2	0.0599 (14)	0.0838 (16)	0.0720 (16)	−0.0015 (11)	0.0120 (11)	−0.0577 (13)
O3	0.0535 (14)	0.0717 (14)	0.0720 (15)	−0.0210 (11)	0.0125 (11)	−0.0453 (12)
N1	0.0337 (13)	0.0449 (13)	0.0311 (13)	−0.0116 (10)	0.0008 (10)	−0.0128 (11)
N2	0.0498 (16)	0.0414 (14)	0.0502 (16)	−0.0020 (12)	0.0023 (12)	−0.0224 (12)
C1	0.0354 (16)	0.0461 (16)	0.0335 (16)	−0.0065 (12)	−0.0019 (12)	−0.0080 (14)
C2	0.0353 (15)	0.0344 (14)	0.0263 (14)	−0.0051 (11)	0.0020 (11)	−0.0065 (12)
C3	0.0347 (15)	0.0345 (14)	0.0338 (16)	−0.0010 (11)	0.0005 (12)	−0.0115 (12)
C4	0.0378 (16)	0.0325 (14)	0.0356 (16)	0.0018 (11)	−0.0004 (12)	−0.0130 (12)
C5	0.0367 (16)	0.0440 (16)	0.0353 (16)	0.0019 (12)	0.0052 (12)	−0.0129 (14)
C6	0.0382 (16)	0.0509 (17)	0.0373 (17)	−0.0085 (13)	0.0054 (13)	−0.0124 (14)
C7	0.0371 (15)	0.0360 (15)	0.0346 (16)	−0.0095 (11)	0.0000 (12)	−0.0096 (13)
C8	0.0311 (14)	0.0408 (15)	0.0273 (14)	−0.0089 (11)	0.0017 (11)	−0.0155 (12)
C9	0.0396 (16)	0.0473 (16)	0.0404 (17)	−0.0125 (12)	−0.0057 (12)	−0.0164 (13)
C10	0.0446 (17)	0.0484 (16)	0.0400 (17)	−0.0156 (13)	−0.0026 (12)	−0.0226 (14)
C11	0.0367 (16)	0.0543 (17)	0.0354 (16)	0.0017 (12)	−0.0083 (12)	−0.0191 (14)
C12	0.0383 (15)	0.0339 (14)	0.0304 (15)	−0.0048 (11)	0.0036 (11)	−0.0088 (12)
C13	0.0364 (16)	0.0469 (16)	0.0432 (17)	−0.0091 (12)	0.0015 (12)	−0.0207 (14)
C14	0.0328 (15)	0.0409 (15)	0.0434 (17)	0.0017 (11)	−0.0094 (12)	−0.0201 (13)
C15	0.0423 (16)	0.0393 (15)	0.0335 (15)	−0.0058 (12)	−0.0080 (12)	−0.0133 (13)
C16	0.0373 (15)	0.0362 (15)	0.0352 (16)	−0.0042 (11)	0.0004 (12)	−0.0125 (13)
C17	0.0523 (18)	0.0364 (15)	0.0411 (17)	−0.0026 (12)	−0.0065 (13)	−0.0177 (13)

### Geometric parameters (Å, º)

**Table:**

O1—C1	1.267 (3)	C9—H9B	0.9900
O2—N2	1.239 (3)	C10—C11	1.525 (3)
O3—N2	1.236 (3)	C10—C17	1.528 (3)
N1—C7	1.289 (3)	C10—H10	1.0000
N1—C8	1.481 (3)	C11—C12	1.520 (3)
N1—H1N	0.95 (3)	C11—H11A	0.9900
N2—C4	1.441 (3)	C11—H11B	0.9900
C1—C6	1.444 (4)	C12—C13	1.528 (3)
C1—C2	1.452 (3)	C12—C16	1.535 (3)
C2—C3	1.385 (3)	C12—H12	1.0000
C2—C7	1.428 (3)	C13—C14	1.525 (3)
C3—C4	1.374 (3)	C13—H13A	0.9900
C3—H3	0.9500	C13—H13B	0.9900
C4—C5	1.406 (3)	C14—C17	1.527 (3)
C5—C6	1.345 (4)	C14—C15	1.539 (3)
C5—H5	0.9500	C14—H14	1.0000
C6—H6	0.9500	C15—H15A	0.9900
C7—H7	0.9500	C15—H15B	0.9900
C8—C9	1.522 (3)	C16—H16A	0.9900
C8—C15	1.528 (3)	C16—H16B	0.9900
C8—C16	1.529 (3)	C17—H17A	0.9900
C9—C10	1.522 (4)	C17—H17B	0.9900
C9—H9A	0.9900		
			
C7—N1—C8	126.4 (2)	C17—C10—H10	109.4
C7—N1—H1N	113.5 (16)	C12—C11—C10	109.2 (2)
C8—N1—H1N	119.8 (16)	C12—C11—H11A	109.8
O3—N2—O2	122.0 (3)	C10—C11—H11A	109.8
O3—N2—C4	119.2 (2)	C12—C11—H11B	109.8
O2—N2—C4	118.8 (2)	C10—C11—H11B	109.8
O1—C1—C6	122.3 (2)	H11A—C11—H11B	108.3
O1—C1—C2	122.3 (2)	C11—C12—C13	109.8 (2)
C6—C1—C2	115.4 (3)	C11—C12—C16	109.7 (2)
C3—C2—C7	118.9 (2)	C13—C12—C16	109.2 (2)
C3—C2—C1	121.0 (2)	C11—C12—H12	109.4
C7—C2—C1	120.1 (2)	C13—C12—H12	109.4
C4—C3—C2	120.3 (2)	C16—C12—H12	109.4
C4—C3—H3	119.9	C14—C13—C12	109.35 (19)
C2—C3—H3	119.9	C14—C13—H13A	109.8
C3—C4—C5	120.6 (3)	C12—C13—H13A	109.8
C3—C4—N2	119.7 (2)	C14—C13—H13B	109.8
C5—C4—N2	119.8 (2)	C12—C13—H13B	109.8
C6—C5—C4	120.6 (3)	H13A—C13—H13B	108.3
C6—C5—H5	119.7	C13—C14—C17	109.3 (2)
C4—C5—H5	119.7	C13—C14—C15	109.8 (2)
C5—C6—C1	122.1 (3)	C17—C14—C15	109.8 (2)
C5—C6—H6	119.0	C13—C14—H14	109.3
C1—C6—H6	119.0	C17—C14—H14	109.3
N1—C7—C2	122.2 (2)	C15—C14—H14	109.3
N1—C7—H7	118.9	C8—C15—C14	108.5 (2)
C2—C7—H7	118.9	C8—C15—H15A	110.0
N1—C8—C9	107.1 (2)	C14—C15—H15A	110.0
N1—C8—C15	111.4 (2)	C8—C15—H15B	110.0
C9—C8—C15	109.8 (2)	C14—C15—H15B	110.0
N1—C8—C16	109.2 (2)	H15A—C15—H15B	108.4
C9—C8—C16	109.9 (2)	C8—C16—C12	109.0 (2)
C15—C8—C16	109.45 (19)	C8—C16—H16A	109.9
C10—C9—C8	109.6 (2)	C12—C16—H16A	109.9
C10—C9—H9A	109.7	C8—C16—H16B	109.9
C8—C9—H9A	109.7	C12—C16—H16B	109.9
C10—C9—H9B	109.7	H16A—C16—H16B	108.3
C8—C9—H9B	109.7	C14—C17—C10	109.3 (2)
H9A—C9—H9B	108.2	C14—C17—H17A	109.8
C9—C10—C11	109.8 (2)	C10—C17—H17A	109.8
C9—C10—C17	109.2 (2)	C14—C17—H17B	109.8
C11—C10—C17	109.7 (2)	C10—C17—H17B	109.8
C9—C10—H10	109.4	H17A—C17—H17B	108.3
C11—C10—H10	109.4		
			
O1—C1—C2—C3	−177.2 (2)	C16—C8—C9—C10	−59.6 (3)
C6—C1—C2—C3	3.0 (3)	C8—C9—C10—C11	59.8 (3)
O1—C1—C2—C7	1.0 (4)	C8—C9—C10—C17	−60.5 (3)
C6—C1—C2—C7	−178.7 (2)	C9—C10—C11—C12	−60.2 (3)
C7—C2—C3—C4	−179.4 (2)	C17—C10—C11—C12	59.8 (3)
C1—C2—C3—C4	−1.2 (3)	C10—C11—C12—C13	−59.8 (3)
C2—C3—C4—C5	−0.5 (4)	C10—C11—C12—C16	60.3 (3)
C2—C3—C4—N2	179.8 (2)	C11—C12—C13—C14	60.2 (3)
O3—N2—C4—C3	2.7 (3)	C16—C12—C13—C14	−60.2 (3)
O2—N2—C4—C3	−177.5 (2)	C12—C13—C14—C17	−60.2 (3)
O3—N2—C4—C5	−176.9 (2)	C12—C13—C14—C15	60.3 (3)
O2—N2—C4—C5	2.9 (3)	N1—C8—C15—C14	−178.32 (19)
C3—C4—C5—C6	0.1 (4)	C9—C8—C15—C14	−59.9 (3)
N2—C4—C5—C6	179.8 (2)	C16—C8—C15—C14	60.8 (3)
C4—C5—C6—C1	2.0 (4)	C13—C14—C15—C8	−60.3 (3)
O1—C1—C6—C5	176.8 (2)	C17—C14—C15—C8	59.8 (2)
C2—C1—C6—C5	−3.5 (4)	N1—C8—C16—C12	176.50 (19)
C8—N1—C7—C2	−176.4 (2)	C9—C8—C16—C12	59.3 (3)
C3—C2—C7—N1	177.8 (2)	C15—C8—C16—C12	−61.3 (3)
C1—C2—C7—N1	−0.4 (4)	C11—C12—C16—C8	−59.8 (3)
C7—N1—C8—C9	−174.7 (2)	C13—C12—C16—C8	60.6 (3)
C7—N1—C8—C15	−54.6 (3)	C13—C14—C17—C10	60.2 (3)
C7—N1—C8—C16	66.4 (3)	C15—C14—C17—C10	−60.3 (3)
N1—C8—C9—C10	−178.11 (19)	C9—C10—C17—C14	60.2 (3)
C15—C8—C9—C10	60.9 (3)	C11—C10—C17—C14	−60.1 (3)

### Hydrogen-bond geometry (Å, º)

**Table:**

*D*—H···*A*	*D*—H	H···*A*	*D*···*A*	*D*—H···*A*
N1—H1*N*···O1	0.95 (3)	1.79 (3)	2.597 (3)	140 (2)
